# Environmental selection of planktonic methanogens in permafrost thaw ponds

**DOI:** 10.1038/srep31312

**Published:** 2016-08-09

**Authors:** Sophie Crevecoeur, Warwick F. Vincent, Connie Lovejoy

**Affiliations:** 1 Département de Biologie, Centre d’études nordiques (CEN) and Takuvik Joint International Laboratory, Université Laval, Québec, QC G1V 0A6, Canada; 2 Institut de Biologie Intégrative et des Systèmes, Université Laval, Québec, QC G1V 0A6, Canada; 3 Québec-Océan, Université Laval, Québec, QC G1V 0A6, Canada

## Abstract

The warming and thermal erosion of ice-containing permafrost results in thaw ponds that are strong emitters of methane to the atmosphere. Here we examined methanogens and other Archaea, in two types of thaw ponds that are formed by the collapse of either permafrost peat mounds (palsas) or mineral soil mounds (lithalsas) in subarctic Quebec, Canada. Using high-throughput sequencing of a hypervariable region of 16S rRNA, we determined the taxonomic structure and diversity of archaeal communities in near-bottom water samples, and analyzed the *mcrA* gene transcripts from two sites. The ponds at all sites were well stratified, with hypoxic or anoxic bottom waters. Their archaeal communities were dominated by Euryarchaeota, specifically taxa in the methanogenic orders Methanomicrobiales and Methanosarcinales, indicating a potentially active community of planktonic methanogens. The order Methanomicrobiales accounted for most of the *mcrA* transcripts in the two ponds. The Archaeal communities differed significantly between the lithalsa and palsa ponds, with higher alpha diversity in the organic-rich palsa ponds, and pronounced differences in community structure. These results indicate the widespread occurrence of planktonic, methane-producing Archaea in thaw ponds, with environmental selection of taxa according to permafrost landscape type.

Archaea are widely distributed throughout the biosphere and play key roles in biogeochemical cycling processes, including methane production^1^. Methanogenic Archaea are responsible for a large fraction of organic carbon decomposition under anaerobic conditions^2^, and their metabolic activities in lakes and natural wetlands may account for more than 30% of total methane emissions to the atmosphere^3^. In high latitude northern regions, diverse archaeal communities have been reported in soils^4^, wetlands^5^, lakes^6^, rivers^7^, and marine environments^8^,^9^. Permafrost landscapes in many parts of the Arctic contain abundant lakes and ponds that have been formed by thawing and collapse of ice-rich soils, and these so-called thaw or thermokarst lakes^4^ are strong emission sources of both CH_4_ and CO_2_^10^. With ongoing climate warming in northern regions, the thawing of permafrost soils may lead to increased mobilisation and transfer of ancient carbon reserves into such thaw ponds, where some of this organic material would be available for decomposition by methanogens. Permafrost thaw lakes and ponds are among the most abundant freshwater ecosystems in the circumpolar North^11^, but studies of archaeal communities in these environments to date have focused on pond sediments in the High Arctic^12^ and wetland soils in the Subarctic^13^, while the presence, diversity, and substrate preferences of methanogens in the water column plankton have not been explored.

Methanogenic Archaea use different pathways for methanogenesis that depend on the carbon substrate and electron donor. The two substrates that are the most commonly used are H_2_/CO_2_ through hydrogenotrophic methanogenesis and acetate using acetoclastic methanogenesis. A third, less common methylotrophic pathway requires the use of a methyl group as substrate^14^. The hydrogenotrophic pathway is found in almost all orders of methanogens (Methanococcales, Methanopyrales, Methanobacteriales, Methanosarcinales, Methanomicrobiales, Methanocellales and the recently discovered Methanomassiliicoccales^14^,^15^), while the genus *Methanosaeta* in the order Methanosarcinales is an obligate acetotroph^16^. Only the order Methanosarcinales has been shown to contain genera or species able to use the three different pathways^14^. Both hydrogenotrophic and acetotrophic taxa of Archaea have been detected in the soil of Subarctic wetlands^13^ and High Arctic thaw ponds^12^.

In the Hudson Bay region of subarctic Québec, thermokarst lakes and ponds have been increasing in size and number over the last three decades in response to rapid warming^17^. These thaw ponds emit CH_4_ to the atmosphere^18^, implying active methanogenic communities. Although there is some evidence of methanogenesis in oxic waters^19^,^20^, methanogenesis by Archaea usually occurs under anoxic conditions^21^, and is especially likely in anoxic sediments. However, the bottom waters of Quebec subarctic thaw ponds are generally hypoxic and sometimes anoxic during summer^18^,^22^,^23^, providing conditions for methanogenic activity. In addition, prolonged anoxia occurs throughout the water column during winter when the ponds are ice covered^24^. Episodic mixing events occur especially during autumn prior to freeze up, but also in spring and occasionally during summer^18^. These mixing events are likely to accelerate the ventilation of CH_4_ into the atmosphere and oxygenate the water column^24^, which would disrupt the redox conditions conducive to anaerobic methanogenesis. The sensitivity of these ponds to mixing raises the question of whether methanogens are active members in the microbial water column plankton.

The substrates available for methanogens in thaw ponds may vary depending on the landscape characteristics of the surrounding catchment. The extent of thawing and associated permafrost erosion will influence the quantity of allochthonous organic matter, while soil properties will influence the nature and lability of the organic matter. In the Quebec subarctic region, most thaw ponds originate from one of two different types of permafrost landscapes; either peatland palsa mounds or mineral lithalsa mounds^25^,^26^. Eroding, carbon-rich palsa soils are likely to release large amounts of organic matter^27^, some of which can be biologically or photochemically broken down^28^ into substrates that could stimulate microbial activity including archaeal methanogenesis.

The aims of the present study were to determine the diversity and environmental partitioning of archaeal communities in subarctic thaw ponds. Specifically, we evaluated the following hypotheses: (1) the bottom waters of these ponds provide a suitable habitat for methanogenic Archaea; (2) permafrost landscape type (peatland palsa versus mineral lithalsa) and the extent of permafrost degradation affect archaeal community structure; and (3), the carbon enriched conditions of the peatland palsa catchment favour greater archaeal diversity compared to mineral lithalsa dominated catchments. We tested these hypotheses using microbial plankton samples taken from ponds in three different permafrost valleys in subarctic Quebec, and the communities were identified using high-throughput sequencing (Illumina MiSeq) of the V6–V8 hypervariable region of archaeal 16S rRNA. We further examined the potential metabolic diversity of methanogenic Archaea by high throughput sequencing of mRNA transcripts of the *mcrA* gene. This gene codes for subunit A of the methyl-coenzyme M reductase and has been used as a proxy for studying taxonomic richness and community composition of methanogens, including in sediments across a palsa wetland gradient in subarctic Norway^13^. Given that many of these thaw ponds contain high concentrations of suspended sediments^18^, we also tested for differences in archaeal taxonomic structure between particle-attached and free-living communities.

## Results

### Limnological conditions

All the ponds were thermally stratified during the period of sampling, with low or near zero values of dissolved oxygen at the bottom (Table 1). Ponds from the KWK (lithalsa) and SAS (palsa) valleys were hypoxic to anoxic at the bottom. Values for pH were higher in the BGR (lithalsa) valley, followed by the KWK valley and then the SAS valley, where the waters were slightly acidic. DOC values tended to be higher for ponds from the SAS valley, although KWK1 and KWK23 also had relatively high concentrations of DOC. The highest TSS values were recorded from KWK1 and KWK23 followed by BGR2. Total phosphorus concentrations were the highest in the KWK ponds and in BGR2.

### Archaeal alpha-diversity

**Figure 1:**
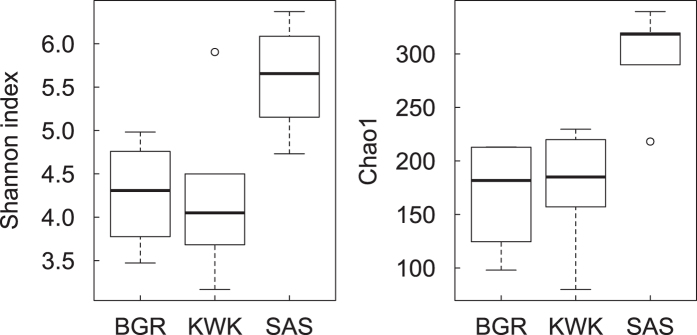
Alpha-diversity measures for the three sampled valleys. Shannon diversity and Chao1 species richness indices are shown for the BGR, KWK and SAS valleys. The line in each box plot indicates the median, the box delimits the 25^th^ and 75^th^ percentile, and the whisker is the range. The diversity indices for the SAS valley differed significantly from the two other valleys (n = 16; P = 0.02 for the Chao1 and P = 0.01 for the Shannon index).

The surface water samples yielded archaeal 16S rRNA sequences from only 3 of the 16 pond samples (SAS2A, SAS2B and BGR2; see Supplementary Table S1), and no *mcrA* transcripts could be amplified from any of these surface samples. We therefore focused our subsequent analyses exclusively on 16 bottom water samples (8 ponds, two size fractions). These yielded a total of 747,149 reads after quality filtering and removal of singletons, and corresponded to 473 OTUs. The cDNA for *mcrA* was successfully amplified and sequenced from two bottom samples: the small (<3-μm) fraction of KWK23 and the large (>3-μm) fraction of SAS2B. The *mcrA* transcripts yielded 112,320 reads corresponding to 142 OTUs. The 16S rRNA rarefaction curve plateau was higher in the SAS than the BGR and KWK ponds, with 200 and 250 OTUs on average for the SAS valley and 100 and 150 OTUs on average for the BGR and KWK valleys (see Supplementary Fig. S1). Some individual SAS samples did not reach a plateau above 250 OTUs, suggesting that these communities may have been under-sampled. ANOVA analysis showed Chao1 (P = 0.02) and Shannon (P = 0.013) indices differed significantly among valleys. Tukey HSD tests showed that SAS differed from BGR (P = 0.04 for the Shannon index and P = 0.03 for the Chao1 index) and KWK (P = 0.003 for the Shannon index and P = 0.01 for the Chao1 index), with no significant difference between KWK and BGR. For the Shannon index, the median in the SAS valley was 5.7 with a range from 4.7 to 6.4, while the medians for BGR and KWK valley were 4.3 and 4.0 respectively, with ranges from 3.5 to 5 and 3.2 to 4.5. For the Chao1 index, the median in the SAS valley was 318 and ranged from 290 to 339. The medians for the BGR and KWK valleys were 182 and 185 respectively, with ranges from 98 to 213 and 80 to 230. In summary, the SAS ponds had higher archaeal diversity and species richness than those in the two other valleys (Fig. 1).

### Archaeal community dissimilarities and composition

**Figure 2:**
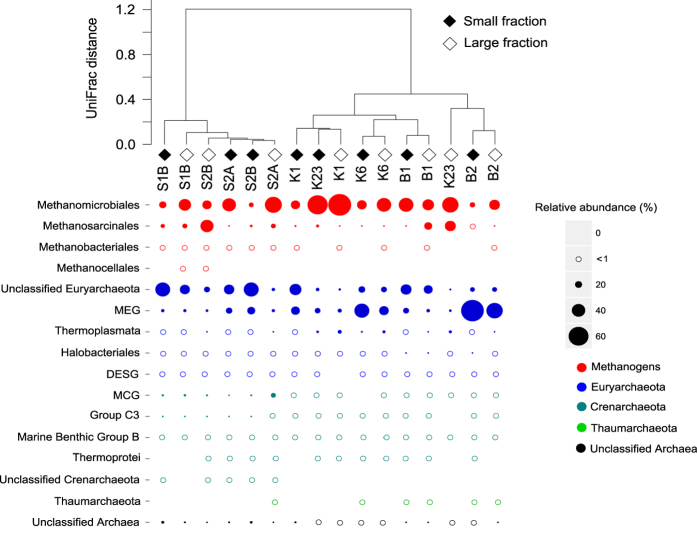
UniFrac clustering and composition of archaeal communities. The upper dendrogram shows the phylogenetic unweighted UniFrac distance among ponds. Filled or open diamonds represent the small and large fractions, respectively. The first letter of each sample name corresponds to the valley name: S for SAS, K for KWK and B for BGR. The following number or combination of letters and numbers indicate the name of the pond. The bubble plot shows the relative abundance of the different archaeal lineages, including the Miscellaneous Euryarchaeotic Group (MEG), Miscellaneous Crenarcheotic Groups (MCG) and Deep Euryarcheotic Sea Group (DESG).

An unweighted UniFrac distance analysis of the bottom water 16S rRNA data showed that the SAS palsa valley clustered apart from the lithalsa valleys KWK and BGR (Fig. 2). A permutation test (9999 permutations) showed that the difference between communities was significant for valleys only (P = 0.001). There were no significant differences between the small and the large fractions and or between the different years of sampling. Pairwise comparisons confirmed that SAS was significantly different from KWK (P = 0.003) and BGR (P = 0.001), while KWK and BGR were not significantly different from each other.

The overall community composition was similar among the bottom water samples (Fig. 2). Methanogens were represented mainly by the order Methanomicrobiales, which composed 13 to 69% of the reads for the large fraction of KWK1. The second dominant group of methanogens was the Methanosarcinales, with a greatest relative abundance of 39% of reads in the large fraction of SAS2B. Two other orders of methanogens (Methanocellales and Methanobacteriales) represented less than 1% of the relative abundance of the total community, with the Methanocellales only found in the SAS valley. The two dominant orders of methanogens, Methanomicrobiales and Methanosarcinales, constituted on average 55% of the KWK valley community, 45% of the SAS valley community and 38% of the BGR valley community. In general, there were more methanogens in the large fraction than in the small fraction, except for KWK23 and BGR1 where Methanomicrobiales were more abundant in the small fraction. The 16S rRNA sequences for the small number of surface water samples similarly contained a high percentage of representatives from the Methanomicrobiales and Methanosarcinales, in both the large and small fractions (Supplementary Table 1).

In addition to the putative methanogenic groups mentioned above, other Euryarchaeota in bottom waters included a large proportion of unclassified Euryarchaea and the Miscellaneous Euryarchaeotic Group (MEG), which accounted for from 6% in the large fraction of SAS2B to 69% of reads in the small fraction of BGR2. In the KWK samples Thermoplasmata were relatively more abundant and represented up to 10% of the reads in the large fraction of KWK1. Finally, Halobacteriales and the Deep Sea Euryarchaeotic Group (DSEG) were found in almost all samples, but represented less than 1% of the reads. Crenarchaeota were also recovered and included the Miscellaneous Crenarchaeotic Group (MCG), which was more common in the SAS valley and represented up to 13% of the reads in the large fraction of SAS2A. Other Crenarchaeota groups (group C3, Thermoprotei and Crenarchaeotic Marine Benthic Group B) were <1% of all reads but were present in all three valleys. Finally, the phylum Thaumarchaeota was present in the two BGR valley ponds, one KWK and one SAS sample. Unclassified Archaea ranged from <1% in the small fraction of KWK23 to 7% of reads for the small fraction of SAS1B (Fig. 2).

**Figure 3:**
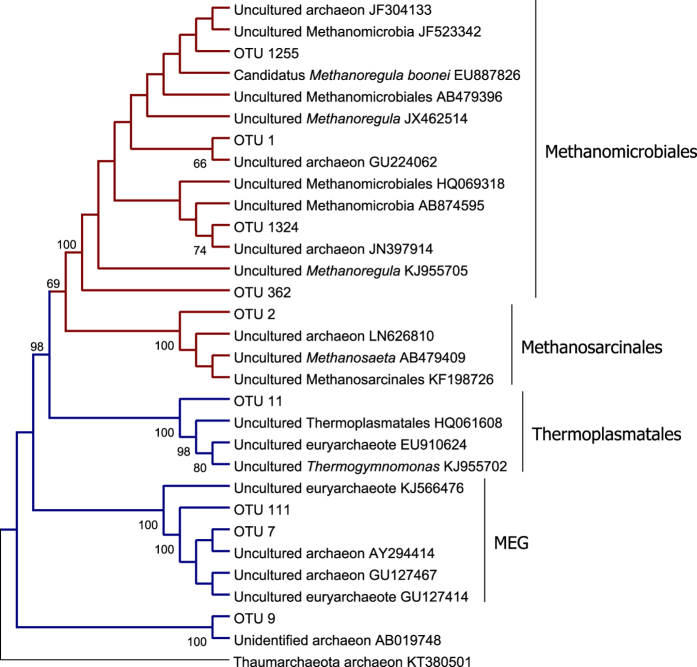
Neighbour-joining phylogenetic tree based on the 16S rRNA sequences for the 5 most abundant OTUs for each valley and their closest matches in GenBank. A Thaumarchaeota archaeon was used as the outgroup. The values at each node represent the percentage bootstrap confidence values using 1,000 replications; only values over 60% are shown. Branches containing methanogenic taxa are in red and for other Euryarchaeota are in blue.

Methanogenic Archaea were consistently among the five most abundant OTUs (defined at a level of 97% similarity) for each valley (Fig. 3). The two most abundant OTUs across all bottom water samples were *Methanoregula* and *Methanosaeta* (Table 2). The KWK and SAS valleys shared another abundant OTU of *Methanoregula* and the two other of the most abundant OTUs in the SAS valley were also in the genus *Methanoregula*. The five most abundant OTUs in the SAS valley were exclusively methanogenic Archaea. Other abundant OTUs in KWK and BGR valleys belonged to the MEG and had high identity percentages with sequences isolated from a petroleum hydrocarbon-contaminated aquifer. Other OTUs in the top five were either unclassified Euryarchaeota (BGR) or Thermoplasmatales (KWK). The most abundant OTUs in the valleys showed no specificity to a particular habitat type with high homology with sequences isolated from diverse freshwater and marine environments.

### Methanogens inferred from the *mcrA* versus 16S rRNA analyses

**Figure 4:**
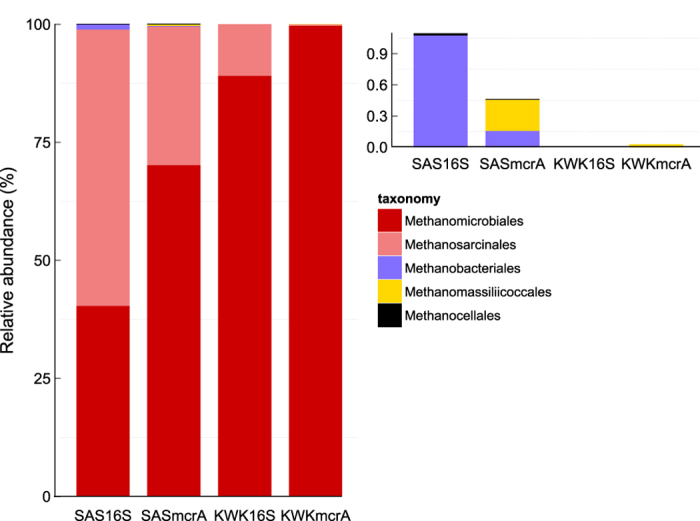
Comparison of the relative abundance of methanogens in the *mcrA* community and the 16S rRNA community. The left-hand plot shows the entire methanogenic community and the right-hand plot shows the groups representing less than 1% of the reads. The SAS sample is the large fraction (3–20 μm) of SAS2B and the KWK sample is the small fraction (0.2–3 μm) of KWK23.

The *mcrA* reads were strongly dominated by the Methanomicrobiales and contained a much lower proportion of other methanogenic groups compared to the 16S rRNA community (Fig. 4). Methanomicrobiales accounted for 70% of *mcrA* reads versus 40% of the 16S rRNA reads in the SAS sample, and 99% of *mcrA* reads versus 89% of 16S rRNA reads in the KWK sample. Conversely, the proportion of Methanosarcinales was lower in the *mcrA* sequences with 30% of *mcrA* versus 58% of 16S rRNA for the SAS sample and 0.3% of the *mcrA* versus 11% of 16S rRNA reads for the KWK sample. The Methanobacteriales and Methanocellales that were recovered at low relative abundance in the 16S rRNA reads were below 1% of reads in the *mcrA* results. The order Methanomassiliicoccales was not detected in the 16S rRNA sequences but was found in the *mcrA* community, with 0.3% of reads for the SAS sample and 0.03% of reads for the KWK samples (Fig. 4).

### Environmental variables and archaeal community clustering

**Figure 5:**
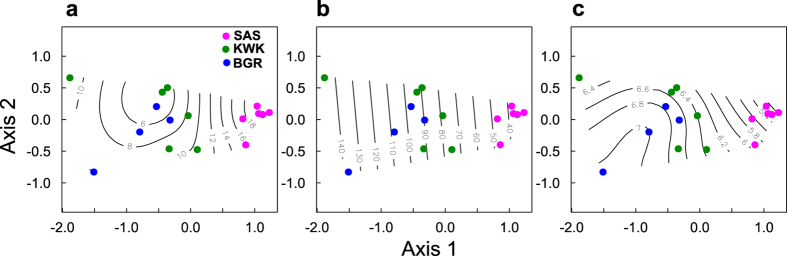
Non metric multidimensional scaling (NMDS) of the community composition. Phylogenetic Unifrac distances are overlaid with environmental variables: (**a**) dissolved organic carbon, (**b**) total phosphorus and (**c**) pH.

NMDS ordination based on the unweighted UniFrac phylogenetic distance was carried out to determine how the measured environmental variables may have influenced archaeal community structure. The two dimensional NMDS had a stress value of 0.085, which indicated good representation of the community arrangement. The ordination of the bottom water communities was significantly correlated with three environmental variables: TP (P = 0.006), DOC (P = 0.008) and pH (P = 0.006). The ordination was consistent with the community dendrogram analysis (Fig. 2), with the SAS valley communities being phylogenetically distinct relative to the two other valleys (Fig. 5). Clustering followed the isopleths of three variables; higher DOC (Fig. 5a) and lower TP (Fig. 5b) and lower pH (Fig. 5c) contributed to the separation of the SAS valley from the other two valleys.

## Discussion

The bottom waters of all of the thaw ponds sampled in the present study harbored Archaea, with a major fraction of the 16S rRNA reads (up to 60%) assigned to methanogenic taxa. These results imply that archaeal methane-producers are likely to occur at relatively high concentration in the microbial plankton and would contribute to the extreme accumulation of methane reported from these ponds: Laurion *et al*.^18^ measured up to 100 μmol CH_4_ L^−1^ at the bottom of KWK23, Deshpande *et al*.^24^ showed that the surface waters of SAS, KWK and BGR ponds were supersaturated in CH_4_ with highest values in a SAS pond, while Matveev *et al*.^29^ found that all of the SAS peatland ponds had high rates of CH_4_ emission to the atmosphere, and 100-fold increases in CH_4_ concentration with depth down their water columns.

In our study, Methanomicrobiales and Methanosarcinales dominated both 16S rRNA and the *mcrA* transcripts. Both orders are widely reported from planktonic environments (see Auguet *et al*.^30^), including boreal lakes^31^. The two orders have also been found in northern peatland soils^4^ and wetlands^13^, implying habitat plasticity. However, these methanogens are normally found in high abundance in environments where oxygen and other electron acceptors are depleted^32^ and methanogenesis is a strictly anaerobic process^21^. In the permafrost thaw ponds sampled here, the methanogens occurred in some bottom waters where oxygen tensions were well above 0% saturation (up to 37% in BGR) and in the aerobic surface waters of three ponds. Methane production has been reported from oxic water^19^,^20^, however the biochemical mechanisms are unresolved. The presence of rRNA-containing methanogens under this wide range of conditions implies the persistence of a viable summer community that may be primed for full activity once the water columns become completely anoxic, which occurs soon after winter freeze-up^24^. There is evidence that Methanomicrobiales in Arctic and peatland soils may be tolerant of variable redox conditions^33^, which may contribute towards their occurrence and success in these subarctic thaw ponds. This group of hydrogenotrophic methanogens may also be favored by the high concentrations of CO_2_ that accumulate in the bottom waters of the permafrost thaw lakes, for example up to 500 and 5400 μmol CO_2_ L^−1^ in the anoxic bottom waters of KWK23^18^ and SAS2A^29^, respectively. Extreme low temperature environments are reported to favor acetotrophic methanogens over hydrogenotrophic taxa, in sediments^34^ and soils^35^, however at least in summer, temperatures in the ponds were within the range that would favor the hydogenotrophic taxa.

In the present study we used RNA as a template and amplified 16S rRNA rather than using DNA, which would amplify the gene only. The rRNA templates reflect more closely Archaea that were actively growing and producing proteins than DNA templates. There are reported inconsistencies, however, between ribosomes (rRNA content) and growth rates, and this approach only provides a measure of potentially active cells^36^. In the BGR ponds, there was an inconsistency between the *mcrA* transcripts and the 16S rRNA, with high proportions of Methanomicrobiales 16S rRNA reads but no successful amplification of the mRNA for *mcrA* as an indicator of active methanogenesis^5^. This may be the result of methodological problems, for example the delay between sampling and filtration because of the remoteness of the BGR valley, and degradation of the messenger RNA. Alternatively it may indicate that viable methanogens were present but that their methane-producing activity was suppressed at the transcriptional level, for example by the ambient oxygen levels that were higher than in the other ponds. Similarly, our detection of 16S rRNA reads for methanogens in the surface samples from a small number of the ponds and the absence of *mcrA* transcripts in these samples would be consistent with evidence from elsewhere that methanogens remain viable in oxygenated waters, but that such conditions inhibit methanogenesis^37^. In the future, it will be useful to make direct measurements of CH_4_ production and loss rates, and to determine the balance between planktonic and benthic methanogenesis in these methane-rich waters.

There was a clear effect of permafrost landscape type (peatland palsa versus mineral lithalsa) on archaeal diversity and community structure. The permutation test on the UniFrac matrix showed that there were no significant differences in archaeal community structure between the two lithalsa valleys that differed in extent of permafrost degradation, from sporadic permafrost in the south (KWK) to discontinuous permafrost in the north (BGR), but that SAS significantly differed from them both. The thawing of the organic-rich peatland would release both particulate and dissolved organic carbon into the pond water, and the SAS communities were well separated, clustering at the upper end of the DOC gradient (Fig. 5a). Soil archaeal communities are strongly shaped by carbon availability and composition^38^, and methanogenic activity is related to organic matter quantity and quality^39^. The degradation of the SAS peatland palsas may result in a not only more carbon but also a greater variety of organic substrates for methanogens compared to the more mineral lithalsa soils. The greater variety of substrates could potentially support the more diverse communities, and the greater proportion of Methanosarcinales. The five most abundant OTUs for the SAS valleys were all methanogenic Archaea, but with different carbon strategies, from the hydrogenotrophic genus *Methanoregula*^39^ to the obligate acetotrophic genus *Methanosaeta*^40^. The newly discovered methanogenic order Methanomasiliicoccales was detected in both ponds by the *mcrA* analysis, but in much higher abundance in SAS than in KWK.The subarctic thaw lakes sampled here contained variable, often high concentrations of suspended solids (Table 1). In systems elsewhere, such particles may influence community structure. For example using the same fractionation protocol as in the present study, Galand *et al*.^7^ reported greater diversity of Archaea in the particle-rich waters of an Arctic river and its receiving coastal waters compared to the adjacent, more oligotrophic marine system. Here, we failed to detect any systematic difference between the two fractions, in either diversity or community structure. This may be due to methodological limitations, with the clogging of the 3 μm filters and retention of the free-living fraction, or may simply reflect a lack of partitioning as a function of particle size. In the more aerobic BGR ponds, particles may offer a refuge to anaerobic methanogens as may occur in the ocean^41^, but there was no evidence of methanogenic enrichment in this fraction.

Other environmental variables that may select for specific archaeal taxa are pH and inorganic nutrients. Archaeal diversity in soil can decrease with increasing pH^42^, in our water samples the SAS valley also had the lowest pH, consistent with such a pattern, despite the narrow pH range between our ponds and sites. Phosphorus availability may also influence diversity or select for certain groups. A study on rice roots indicated that high phosphate concentrations inhibit members of the family Methanosarcinaceae and favored Methanobacteriaceae^43^, but the latter group was present only in low abundance in the thaw ponds.

In conclusion, Euryarchaeota, were found in the microbial plankton of thaw ponds and had a high proportion of the methanogenic orders Methanobacteriales and Methanosarcinales. The *mcrA* reads indicated that the Methanomicrobiales dominated, and pointed to the importance of the hydrogenotrophic pathway for methanogenesis in these waters. There was a distinct separation between palsa and lithalsa sites, suggesting that the greater supply and diversity of carbon substrates in the palsa ponds selected for a significantly different, more diverse archaeal community than in the lithalsa ponds. These results imply that permafrost landscape type exerts a strong environmental filtering effect on archaeal community structure in these northern aquatic environments.

## Methods

### Study site and sampling

Samples were collected 1 to 13 August 2012 and 31 July to 19 August 2013 from three different valleys on the eastern side of Hudson Bay, in northern Quebec, Canada. The KWK (55^◦^16′N; 77^◦^46′W) and SAS (55^◦^13′N; 77^◦^42′W) valleys are located near the village of Whapmagoostui-Kuujjuarapik, in a region of sporadic permafrost where permafrost covers less than 2% of the landscape. The BGR valley (56^◦^37′N; 76^◦^13′W) is situated 100 km north of the two other valleys, in the discontinuous permafrost region, close to the village of Umiujaq. The SAS valley is covered in peatland and ponds from the thawing of organic palsas^44^, while BGR and KWK ponds originated from lithalsas^26^. Two to three ponds were selected from each valley: BGR 1 and BGR 2, KWK 1, 6, 23 and SAS 1B, 2A and 2B. Pond number and names were chosen to be consistent with previous literature for this region^18^,^22^,^26^,^45^,^46^. All of the sites were accessed by helicopter and the ponds were sampled from an inflatable boat positioned over the central region of maximum depth. Profiles of temperature, dissolved oxygen (DO), and pH were taken with a 600R multiparametric probe (Yellow Spring Instrument). Bottom samples of the microbial plankton communities were collected using a horizontally mounted Van Dorn bottle (Wilco) positioned 0.5 m above the sediments. The water was immediately transferred to acid-washed, 4-L Cubitainers™ that were rinsed three times with sample water prior to filling, and were overfilled to avoid oxygenation Surface samples were also collected in rinsed Cubitainers, held 0.2 m beneath the pond surface. All Cubtainers were capped and placed in coolers after filling, and were returned to the laboratory by helicopter for immediate filtration.

### Physico-chemical and molecular analysis

Water samples for physico-chemical analysis (DOC, TSS and TP) and for molecular analysis (RNA) were processed as in Crevecoeur *et al*.^45^. The samples for nucleic acids were extracted with the AllPrep DNA/RNAMini Kit (Qiagen) modified to include an additional step using polyvinylpyrrolidone (PVP, Alfa Aesar) to minimize potential PCR inhibition. This protocol included a DNAse step. The absence of DNA was confirmed by a test PCR using universal 16S primers on the final RNA extract. RNA was converted to cDNA using the High Capacity cDNA Reverse Transcription Kit (Applied Biosystems-Ambion). Amplification of the V6-V8 region of 16S rRNA and *mcrA* was performed using the sequence specific regions described respectively in Comeau *et al*.^9^ and Luton *et al*.^47^ using a two-step dual-indexed PCR approach modified for Illumina instruments. In a first step, the gene specific portion was fused to the Illumina TruSeq sequencing primers (Supplementary Table S2) and PCR was carried out in a total volume of 25 μL that contained HF buffer 1X (NEB), 0.25 μM of each primer, 200 μM of each dNTPs (Life Technology), dimethylsulfoxide (DMSO, NEB) at a final concentration of 3%, 1 U of Phusion High-Fidelity DNA polymerase (NEB) and 1 μL of template cDNA. To decrease potential primer bias, two more reactions with 5 and 10 fold diluted template were also carried out for each sample. Temperature and duration of thermal cycling were started with an initial denaturation at 98 °C for 30 s followed by 40 cycles of denaturation at 98 °C for 10 s, annealing at 55 °C for 10 s, extension at 72 °C for 30 s and a final extension at 72 °C for 300 s. The three dilutions reaction were pooled together and purified using the Axygen PCR cleanup kit (Axygen). Quality and quantity of the purified PCR product were checked on a 1% agarose gel. Fifty to 100 fold dilution of this purified product was used as a template for a second PCR step with the goal of adding barcodes (dual-indexed) and missing sequence required for Illumina sequencing (Supplementary Table S2). This second PCR was done in triplicates under the same conditions as the first PCR but with 13 cycles. Triplicates were pooled together and purified as above and then quantified spectrophotometrically with the Nanodrop 1000 (Thermo Fisher Scientific). Barcoded amplicons were pooled in equimolar concentration for sequencing on the Ilumina MiSeq at the Plateforme d’Analyses Génomiques (IBIS, Université Laval, Québec, Canada). The primers used in this work contain Illumina specific sequences protected by intellectual property rights (Oligonucleotide sequences © 2007-2013 Illumina, Inc.). The raw Illumina sequences have been deposited in the Short Read Archive database under bioproject PRJNA306515.

### Bioinformatic analysis

Sequences of 16S rRNA were analysed using the UPARSE pipeline for Illumina paired-end reads^48^ involving merging pair-ends, quality filtering with a 1.0 expected error rate, dereplication of sequences, sorting sequences by size, removing singletons, clustering OTUs at ≥97%, discarding chimeras, indexing OTUs names and creating OTU tables. The downstream analyses were done within the Qiime pipeline^49^. Taxonomic assignment of these OTUs was performed using the mothur classifier^50^ with a 0.8 confidence threshold based on the SILVA reference database^51^ modified to include sequences from our curated 16S rRNA gene sequence database^52^. Amplicons of *mcrA* were analysed with the FunGene pipeline of RDP server (http://fungene.cme.msu.edu/FunGenePipeline/)^53^. Reads shorter than 150 nt were discarded and chimeras were checked and removed with UCHIME. Sequences were translated and compared to the *mcrA* reference sequence with FrameBot for correcting frameshift errors, sequences having in-frame stop codons were discarded. Amino acid sequences were aligned with HMMER3 and then clustered at 84% similarity that corresponds to OTU level of 97% for 16S rRNA^54^. A custom *mcrA* database was constructed by downloading *mcrA* sequences from the Functional Gene Repository v.8.0 with score no lower than the HMM training sequences. Reference sequences were checked against the NCBI nr database and the in house database was manually curated to ensure all families of methanogens were represented. Taxonomic affiliation of the representative sequences of *mcrA* OTUs was defined by alignment with the reference database using the BLASTp algorithm. A Neighbour Joining (1000 bootstrap) tree following the Poisson model was constructed to assign taxonomy to sequences hitting uncultured methanogens. Most archaeal diversity is associated with uncultured taxa, and the reference database is much less developed than for Bacteria. It is not possible to reliably classify the taxa at the genus level and we therefore adopted the more conservative and reliable cut-off at the order level.

Shannon and Chao1 diversity indexes for the 16S rRNA were estimated using the command alpha_diversity.py available in Qiime. ANOVA assumptions were tested and verified using the Shapiro-Wilk test for normality and the Bartlett test for homoscedasticity. Then three-way analysis of variance (three-way ANOVA) was used to assess differences in diversity indexes between valleys, fractions, and year. An a posteriori Tukey HSD test was run to identify differences between valleys. For beta-diversity analyses, OTU tables were subsampled 100 times at 22,200 reads for 16S rRNA and 30,800 reads for *mcrA*. This number of reads corresponds each time to the smallest number of reads per sample minus 10%. A tree for the 16S rRNA was constructed in Qiime using the method Fasttree in order to calculate the unweighted Unifrac phylogenetic distance^55^ using the command beta_diversity.py.

Further statistical analyses were done with R^56^ (version 3.0.1) and Qiime. A non-metric multidimensional scaling (NMDS) with the UniFrac distance was also used to visualize the influence of the environmental variables on the community. The NMDS was performed in R using the function monoMDS with a principal coordinates analysis done with the function wcmdscale as a starting configuration. Correlations between the environmental variables and the ordination were tested in R with the envfit function. The selected significant environmental variables were then plotted on the ordination using the ordisurf function. These functions were employed via the vegan package (Oksanen *et al*. (2013). Vegan: Community Ecology Package. R package version 2.0-8. http://CRAN.R-project.org/package=vegan; last accessed January 2016). Statistical significance of difference between communities was assessed using permutation test adonis on the UniFrac distance matrix under the function compare_category.py. Pairwise comparisons were then processed using the function make_distance_boxplot.py. The resulting p-values were corrected with Bonferroni. Both of these commands are available in Qiime^49^. The five most abundant OTUs for each valley were subsequently submitted to a BLASTn against the GenBank nr database to assess identification and isolation source of the closest matches. A phylogenetic tree was constructed with these OTUs and closest matches in Genbank. Sequences were aligned using MAFFT version 7 and then checked manually. Models for tree construction were tested with the maximum likelihood algorithm, and the Kimura 2+ Gamma distributed model was selected to construct a neighbour joining 1000 bootstrap tree using MEGA 6.

## Additional Information

**How to cite this article**: Crevecoeur, S. *et al*. Environmental selection of planktonic methanogens in permafrost thaw ponds. *Sci. Rep.* **6**, 31312; doi: 10.1038/srep31312 (2016).

## Supplementary Material

Supplementary Information

## Figures and Tables

**Table 1:** Limnological properties of the bottom water (0.5 m above the sediment) for the sampled thaw ponds.

Pond	Year	T°C	O_2_ (mg L^−1^)	O_2_ (% sat.)	pH	DOC (mg L^−1^)	TSS (mg L^−1^)	TP (μg L^−1^)
BGR1	2013	9.7	3.0	37.1	7.3	2.7	10.4	19.3
BGR2	2012	11.0	3.5	32.7	7.2	8.7	57.4	148.9
KWK1	2012	6.4	0.5	4.2	6.2	12.0	140.8	87.8
KWK6	2012	8.2	1.8	17.5	6.3	5.2	16.0	99.9
KWK23	2012	4.4	0.4	2.7	6.1	10.9	73.6	170.5
SAS1B	2013	11.7	1.0	14.2	6.2	16.2	18.4	26.1
SAS2A	2012	4.6	0.3	2.0	5.6	18.9	16.2	41.5
SAS2B	2012	5.7	0.5	3.8	4.5	21.5	7.7	25.8

Temperature (T °C), dissolved oxygen (O_2_) concentration and O_2_ as % air equilibrium (% sat) dissolved organic carbon (DOC), total suspended solids (TSS) and total phosphorus (TP).

**Table 2:** Identity of the 5 most abundant OTUs (based on 16S rRNA sequences) for each valley following the lowest taxonomic level of the SILVA modified database^52^.

Number of reads	OTU	Valley	SILVA taxonomy	% identity	Accession number	Genbank taxonomy	Isolation source
68710	1	KWK (22%)	*Methanoregula*	100	GU224062	uncultured archaeon	lake water
		BGR (14%)					
		SAS (11%)					
44091	2	SAS (12%)	*Methanosaeta*	100	LN626810	uncultured archaeon	Marine bioreactor
		KWK (8%)					
		BGR (6%)					
24882	1324	KWK (8%)	*Methanoregula*	99	JN397914	uncultured archaeon	spring pit
		SAS (3%)					
22248	7	KWK (10%)	MEG	97	GU127467	uncultured archaeon	reservoir anoxic water
		BGR (9%)					
20494	111	BGR (14%)	MEG	98	AY294414	uncultured archaeon	hydrocarbon-contaminated aquifer
19308	362	SAS (4%)	*Methanoregula*	98	KJ955705	uncultured *Methanoregula*	hydrocarbon-contaminated sediment
17934	9	BGR (8%)	Euryarchaeota	90	AB019748	uncultured archaeon	deep-sea hydrothermal vent
10585	1255	SAS (3%)	*Methanoregula*	98	JF304133	uncultured archaeon	outfall sediment
9430	11	KWK (6%)	Thermoplasmatales	93	EU910624	uncultured euryarchaeote	sediment

OTU refers to the taxon identity numbers in Fig. 3. The groups Euryarchaeota, Miscellaneous Euryarchaeotic Group (MEG) and Thermoplasmatales could not be further assigned. Percent (%) is the proportion of each OTU expressed as a percentage of the total 16S rRNA reads for the archaeal communities in each valley.
