# Supplementary material for: Environmental selection of planktonic methanogens in permafrost thaw ponds
Source: Sci Rep. 2016 Aug 9;6:31312. doi: 10.1038/srep31312 (PMC4977513; doi:10.1038/srep31312)
Supplement: Supplementary Information [file srep31312-s1.pdf]

Supplementary Information

For:

Environmental selection of planktonic methanogens in permafrost thaw ponds

by Crevecoeur S., Vincent W.F. and Lovejoy C.

Supplementary Figure S1

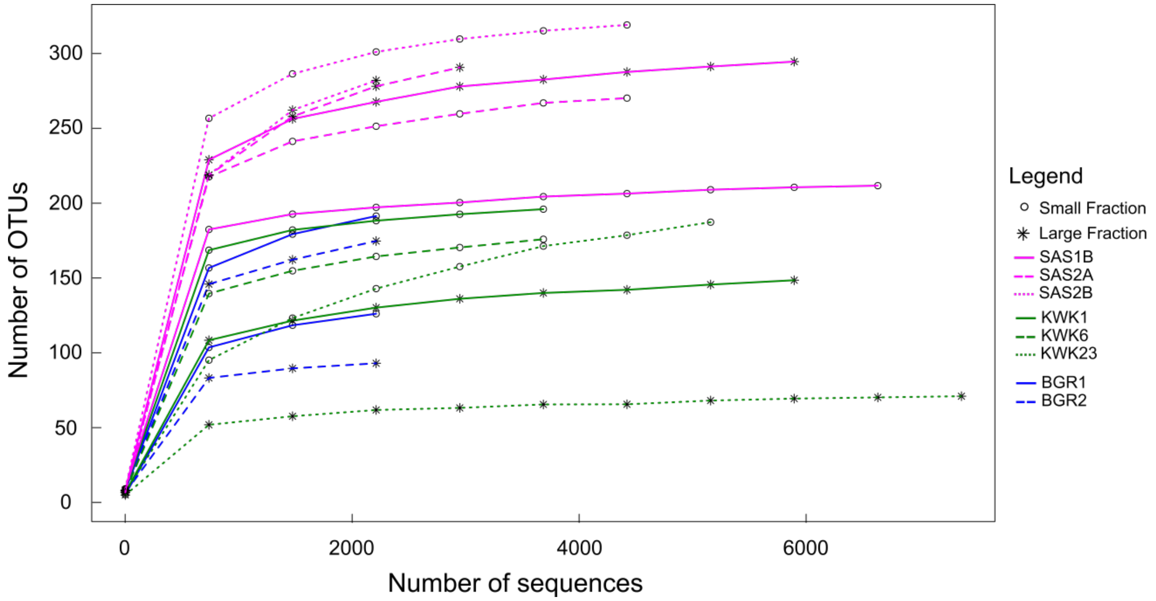

Supplementary Figure S1. Rarefaction curve of the 16S rRNA. OTUs were clustered at 97%.

Supplementary Table S1. 16S rRNA and methanogenic orders recovered from the surface water samples, and percent representation in the sequences.

Taxonomy	SAS 2A		SAS 2B		BGR2	
	Large	Small	Large	Small	Large	Small
Methanomicrobiales	23	4	8	2	51	41
Methanosarcinales	18	< 1	33	<1	8	<1
Methanobacteriales	2	0	1	<1	2	<1
Methanocellales	<1	0	0	0	<1	<1
Unclassified Euryarchaeota	40	69	41	64	23	36
MEG	7	21	12	26	9	18
Thermoplasmata	4	< 1	< 1	< 1	4	< 1
Halobacteriales	< 1	< 1	<1	<1	<1	2
DESG	< 1	0	< 1	2	< 1	< 1
MCG	2	< 1	1	< 1	< 1	0
Group C3	< 1	< 1	< 1	1	< 1	< 1
Marine Benthic Group B	0	< 1	< 1	0	< 1	< 1
Thermoprotei	0	0	0	0	< 1	< 1
Unclassified Crenarchaeota	0	0	< 1	0	< 1	0
Thaumarchaeota	0	0	0	0	0	0
Unclassified Archaea	3	3	3	4	1	1

Supplementary Table S2. Properties of the V6-V8 16S rRNA and mcrA primers fused with the TrueSeq sequencing primers.

Primer	Targeting region	Trueseq primers	References
A-956F	V6-V8	ACACTCTTTCCCTACACGACGCTCTTCCGATCT-TYAATYGGANTCAACRCC	Comeau et al., 2011
A-1401R	V6-V8	GTGACTGGAGTTCAGACGTGTGCTCTTCCGATCT-CRGTGWGTRCAAGGRGCA	Comeau et al., 2011
MLF	*mcrA*	ACACTCTTTCCCTACACGACGCTCTTCCGATCT-GGTGGTGTMGGDTTCACMCARTA	Luton et al., 2002
MLR	*mcrA*	GTGACTGGAGTTCAGACGTGTGCTCTTCCGATCT-TTCATTGCRTAGTTWGGRTAGTT	Luton et al., 2002
Generic forward second-PCR primer		AATGATACGGCGACCACCGAGATCTACAC[index1]ACACTCTTTCCCTACACGAC	
Generic reverse second-PCR primer		CAAGCAGAAGACGGCATACGAGAT[index2]GTGACTGGAGTTCAGACGTGT	
